# Distinct Visual Pathways of Threat Retrieval in Fear‐Conditioned Faces

**DOI:** 10.1111/psyp.70429

**Published:** 2026-10-14

**Authors:** Enya M. Weidner, Marlene Götze, Adrian Taday, Johanna Kissler

**Affiliations:** ^1^ Department of Psychology Bielefeld University Bielefeld Germany

**Keywords:** EEG, fear conditioning, magnocellular pathway, P1, SCR, spatial frequency

## Abstract

Many have demonstrated rapid (< 100 ms) visuo‐cortical differentiation of threat‐associated faces. This may go back to low‐spatial frequency (LSF) visual information originating from magnocellular pathways. Yet it remains unknown whether potentially magnocellular fear signals extend beyond evolutionarily prepared emotional faces. If so, spatial frequency characteristics should modulate visual processing of faces with newly acquired threat‐relevance. Furthermore, it is unclear whether sub‐bands of the visual spectrum are associated with autonomic arousal. Using a differential fear‐conditioning paradigm (*n* = 68), this study tested whether early visual attentional capture, indicated by the P1 event‐related potential component, face encoding, indicated by the N1 component, and autonomic arousal, indicated by skin conductance responses (SCR), prioritizes LSF information of threat‐associated faces with neutral expressions during fear retrieval. Additionally, correlations tested whether the same subcortical system underlies the P1 and SCR. Stimulus ratings and N1 amplitudes confirmed successful fear acquisition. During fear retrieval, the P1 showed a selective left‐hemispheric enhancement in response to LSF threat‐faces. By contrast, the N1 was sensitive to spatial frequency but not fear‐relevance. CS‐differentiation in the SCR was not modulated by spatial frequency but longer CS presentations resulting in larger fear differentiation. Amplitudes of P1 and SCR were positively correlated only for safety faces. Data support the notion that magnocellular‐cortical pathways adapt quickly to novel threat‐associations and facilitate rapid threat retrieval in early visual attention, but not face encoding, even for perceptually neutral faces. At least in the present paradigm, subcortical fear pathways do not associate with anticipatory arousal in SCR.

## Impact Statement

Our electroencephalography (EEG) study provides evidence for distinct, hemisphere‐specific contributions of magno‐ and parvocellular pathways during early visual attention towards fear‐conditioned faces (P1), while fear‐specific face encoding (N1) may depend on the convergence of both pathways. Skin conductance responses reflect anticipatory arousal irrespective of the visual pathway. Together, these results reveal parallel but dissociable mechanisms of fear perception that are differentially sensitive to visual properties of newly threat‐associated faces.

## 1 Introduction

Rapid (< 100 ms) visual attentional engagement with threat‐relevant faces has often been interpreted as evidence for a neural shortcut of fear (Carretié et al. 2017; Garvert et al. 2014; LeDoux 2007; Morris et al. 1999). Presumably, coarse low spatial frequency (LSF) information transmitted by magnocellular subdivisions of the lateral geniculate nucleus suffices to amplify visuocortical responses to threat‐related faces (Vuilleumier et al. 2003; Winston et al. 2003). This would bypass slower parvocellular processing of high spatial frequencies (HSF) (Crook et al. 1988; Maunsell and Gibson 1992; McFadyen et al. 2019; Purpura et al. 1988; Schiller and Malpeli 1978). Still, the relative contribution of these pathways to threat differentiation in early visual perception remains debated (Boatman and Kim 2006; Li and Keil 2023; McFadyen et al. 2017).

Favoring the idea of magnocellular contributions to early visual perception, the P1 event‐related potential (ERP) component, a common marker for early (~90–130 ms) visual attentional capture in electroencephalogram (EEG) studies (Olofsson et al. 2008), has been shown amplified in response to LSF relative to HSF images (de Cesarei et al. 2013) and faces (Nakashima et al. 2008). Crucially, some found P1 amplitudes to differentiate fearful from neutral faces only when LSF spectra of fearful faces were preserved Bruchmann et al. (2020). By contrast, contributions of specific visual pathways to facial threat differentiation in the N1 ERP component (Schindler et al. 2023)—a well‐established marker for structural encoding of faces (Eimer 2011; Hinojosa et al. 2015)—are still debated. Some point towards a convergence of magno‐ and parvocellular signals during N1 threat detection (Bruchmann et al. 2020; Pourtois, Dan, et al. 2005), provided a typical facial configuration is present (Rossion and Caharel 2011; Schindler, Bruchmann, et al. 2021), while others suggest that some facial expressions benefit from specific spatial‐frequency information for N1 modulation (Flevaris et al. 2008; Nakashima et al. 2008; Tian et al. 2018; Vlamings et al. 2009; You and Li 2016).

Hence, what remains particularly unclear is whether LSF threat‐associated faces would be so readily attended to because their stereotypical configuration in LSF (Barrett 2018; Bruchmann et al. 2020; Goffaux et al. 2005; Tipples 2007) enhances salience more strongly than the actual concept of threat. Correspondingly, fear‐neutral differentiation in the P1 was found preserved even for scrambled faces (Schindler, Wolf, et al. 2021). By contrast, Yu et al. (2023) argue that conceptual threat relevance but not perceptual information modulates both the P1 and N1: although observed for upright faces, they found no ERP enhancement for inverted angry versus neutral faces containing the same physical information. However, the different sets of facial expressions used across studies might limit their comparability (N'Diaye et al. 2009; Springer et al. 2007). Understanding the neural pathways that underlie visuocortical threat‐detection and their sensitivity to sub‐bands of the visual spectrum might help to align these inconsistencies.

Fear conditioning can be used to robustly manipulate acquisition of novel conceptual threat‐relevance without confounding perceptual differences (Lonsdorf and Kalisch 2011; Stolarova et al. 2006). Many studies have already reported early‐onset (~100 ms) increases of occipito‐temporal electrophysiological responses, including neural generators of the P1 and N1, to neutral faces associated with an aversive outcome (Bruchmann et al. 2023; Junghöfer et al. 2017; Linton and Levita 2021; Moratti et al. 2006; Roesmann et al. 2020; Sperl et al. 2021; Steinberg et al. 2012). Early effects of fear conditioning have also been found to spatially extend to frontal and parietal regions (Hintze et al. 2014; Petro et al. 2017; Rehbein et al. 2014, 2015; Roesmann et al. 2022; Steinberg et al. 2012), suggesting a parallel engagement of multiple neural pathways during early threat perception. Despite this body of research, to the best of our knowledge, fear conditioning has not been utilized to compare magnocellular and parvocellular contributions to the visual processing of newly acquired facial threat.

In addition, it remains largely unexplored whether distinct visual pathways of threat processing are related to autonomic arousal. Prior functional magnetic resonance imaging (fMRI) (Büchel and Dolan 2000; Büchel et al. 1999, 1998) as well as EEG studies (Antov et al. 2020; Gruss and Keil 2019; Pastor et al. 2015; Stegmann et al. 2024) have repeatedly shown that the skin conductance response (SCR) tracks fear learning in parallel to sensory, frontal, and medial temporal brain areas. Particularly, the magnocellular route is thought to modulate both early visual attention and autonomic output, the latter assumably via the amygdala (Hao et al. 2025; Méndez‐Bértolo et al. 2016; Vuilleumier et al. 2003). Given that P1 amplitudes were previously associated with arousal coding (Key et al. 2005) and early visual perception, as operationalized by steady‐state visual evoked potentials, has been linked to the SCR (Keil et al. 2008), specifically the P1 and SCR may reflect processing of the same magnocellular fear signals through separate efferent channels. Highlighting the role of SF information for fear retrieval in the SCR, Chen et al. (2023) show that fear‐conditioned SCR to chromatic grating stimuli was partly mediated by LSF and conscious perception: For unconsciously perceived gratings, SCR fear differentiation was present for LSF but not HSF stimuli following fear acquisition based on unfiltered stimuli, while both LSF and HSF gratings elicited differential fear responses when consciously perceived. Magnocellular pathways may therefore be able to support autonomic responding even in the absence of conscious perception (Morris et al. 1999). On the other hand, SF effects in the slow, long‐latency SCR might be masked if visual input can be thoroughly evaluated (Baeuchl et al. 2019; Sevenster et al. 2014; Tanskanen et al. 2007).

Against this background, we investigated whether processing of fear‐conditioned faces in the P1 and SCR is facilitated by specific sub‐bands of the visual spectrum. While EEG and SCR were recorded, greyscale photographs of broadband (unfiltered), neutral facial expressions were either fear‐ or safety‐conditioned (Junghöfer et al. 2017; Moratti et al. 2006; Roesmann et al. 2020; Sperl et al. 2021; Steinberg et al. 2012). SF‐specific threat retrieval was subsequently probed by presenting LSF‐ and HSF‐filtered versions of the conditioned faces. Our preregistered hypotheses (https://osf.io/5kbmn/) state that the P1 (Bruchmann et al. 2020) and SCR (Chen et al. 2023) should show highest amplitudes for threat‐associated faces that are shown in LSF. For the N1, we expected generally larger amplitudes for threat‐associated faces compared to neutral faces, without modulations by spatial frequency (Bruchmann et al. 2020; Pourtois, Dan, et al. 2005). Since P1 and SCR are potentially driven by shared magnocellular sources, we also expected the highest correlation of P1 and SCR amplitudes in response to LSF faces. Furthermore, stimulus presentation time was varied randomly (100/1000 ms) to test how the duration of visual processing affects fear differentiation in the SCR. We expected stronger SCR threat differentiation for longer stimulus durations (Sevenster et al. 2014; Tanskanen et al. 2007).

## 2 Method

### 2.1 Sample

The initial preregistered target sample size was 40 participants. Due to a defect of the SCR electrodes, SCR data were not recorded for the first 37 participants. Data collection was therefore extended until our preregistered goal of *n* = 40 was met for the SCR data, resulting in a larger overall sample (*n* = 77). Note that eight participants had already been tested at the time of preregistration, but no data analysis was performed prior to preregistration. No participant reported any psychiatric or neurological disorders, nor the use of drugs in the two weeks prior to testing. Data from four participants were excluded due to poor recording quality and five subjects had excessive artefactual EEG signals. The final sample for the EEG analysis comprised 68 participants (age: M = 25.56 years, SD = 7.27 years; 71.01% female), of whom 58 reported to be right‐handed. Due to the considerable loss of SCR data, we conducted additional EEG analyses including only participants for whom both measures were available. A priori power analysis was not conducted because the study employed nonparametric permutation‐based inference of multilevel data. Therefore, data distribution was not estimated beforehand (Manley 2020; Noguchi et al. 2021; Raychaudhuri 2008). Post hoc power analyses of the decisive effects are reported in the results section. Participants received either €10 or one study credit per hour. The study was conducted in accordance with the Declaration of Helsinki and was approved by the Ethics committee of Bielefeld University (EUB‐2024_245), following the ethics guidelines of the German Psychological Association (DGPs).

### 2.2 Stimuli

We presented neutral facial expressions of four Caucasian male identities (conditioned stimulus, [CS]) from the FACES database (Ebner et al. 2010) with previous permission. Faces were rendered equiluminescent to each other. The isolated, elliptically cropped faces without visible hair, shown in greyscale, were centrally presented on a black screen, measuring 8 cm in width and 12 cm in height (9° 47′ 0.89″ degree visual angle [centered], Figure 1a). Stimuli were presented on a DELL Precision 3650 computer with an ASUS VG24BQE Full HD screen using PsychoPy (version 2024.1.5). The aversive unconditioned stimulus (US) was operationalized as a 1 s white noise burst (90–95 dB). It was presented binaurally via stereo Creative 265 loudspeakers. The volume was configured with an audiometer. During the acquisition phase, broadband versions of the faces were presented. For the retrieval phase, CS were spatially filtered and presented either as LSF (≤ 10 cycles/image; ~0.034 cycles/pixel horizontally, ~0.023 cycles/pixel vertically) or HSF images (≥ 35 cycles/image; ~0.120 cycles/pixel horizontally, ~0.080 cycles/pixel vertically). SF characteristics were chosen based on reported SF sensitivities of magno‐ and parvocellular pathways (Lynch et al. 1992; Merigan and Katz 1990) as well as previous studies on SF‐specific face perception (Bruchmann et al. 2020; Méndez‐Bértolo et al. 2016) and extracted using custom MATLAB scripts (version 2019b, The MathWorks Inc.).

**FIGURE 1:**
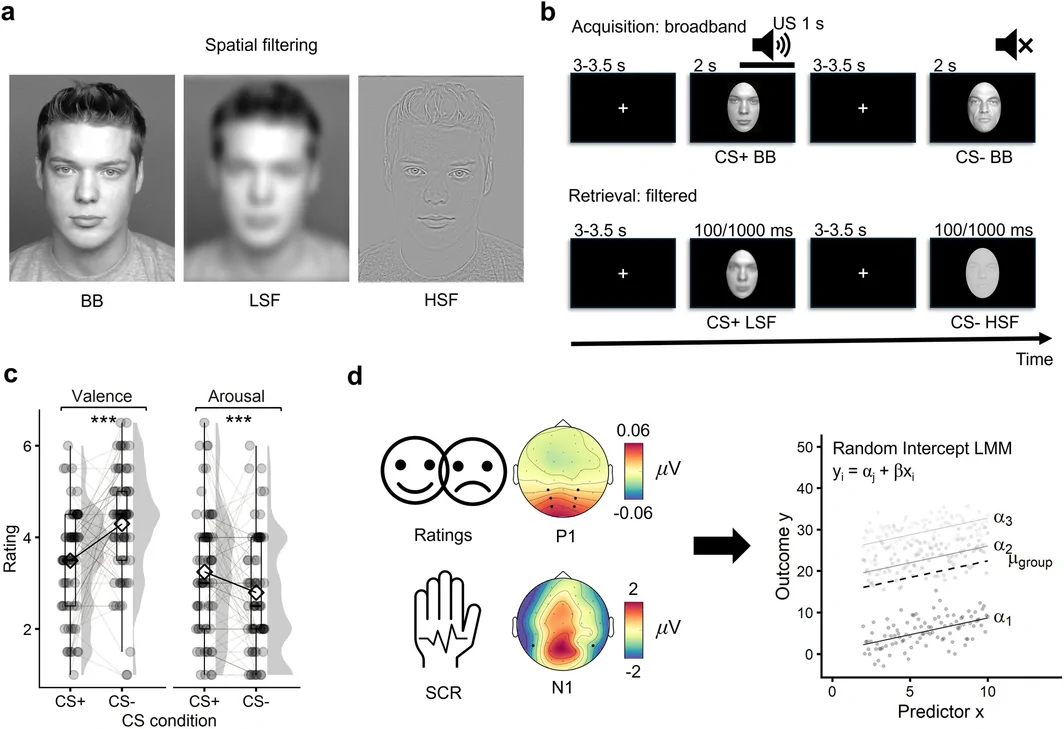
Experimental paradigm and validation of threat‐induction. (a) Examples of broadband (BB) images and low spatial frequency (LSF) or high spatial frequency (HSF) filtering that was applied to the experimental stimuli. Note that for the experiment, faces were cropped (see b). (b) Exemplary trial in the acquisition and the retrieval phase. Please note that the faces shown were not used in the experiment but selected to comply with publishing guidelines of the FACES database. (c) Stimulus ratings of valence and arousal. Boxplots show median and interquartile range; raincloud plots display single‐subject means per CS condition along with the smoothed distribution density; diamonds indicate the overall condition mean. Line plots depict data trends. The black line shows the mean across all subjects; gray lines show single‐subject trends. Brackets mark significant effects of CS on ratings (***p ≤ 0.001). (d) Dependent variables and illustration of the analysis approach. The topography shows mean ERP amplitudes in the P1 and N1 time‐window. The P1 was extracted from 90 to 130 ms after stimulus onset. The N1 was extracted from 140 to 190 ms for the habituation and acquisition phase and from 150 to 210 ms in the retrieval phase. Data were extracted from the channels marked in black. SCR was measured as the first‐interval response in a 900–4500 ms trough‐to‐peak interval after stimulus onset. A linear mixed model (hierarchical regression) was implemented for all measures of interest. BΒ, broadband; CS, conditioned stimulus; HSF, high spatial frequency; LMM, linear mixed model; LSF, low spatial frequency; SCR, skin conductance response; US, unconditioned stimulus.

### 2.3 Procedure

Before the experiment, participants were asked to give their informed consent. Demographic as well as psychiatric variables were documented. Psychiatric assessments included the Beck‐Depression‐Inventory (BDI; M = 6.71, SD = 6.70; Beck et al. 1996), the State–Trait‐Anxiety Inventory (STAI; M_State_ = 32.89, SD_State_ = 6.15, M_Trait_ = 37.55, SD_Trait_ = 8.25; Spielberger 1970), and the Harvard Trauma Questionnaire (HTQ; M_self‐experienced_ = 0.81 event, SD_self‐experienced_ = 1.72 events; Kleijn et al. 2001). Then, the EEG headcap, electrodes, and electrolyte gel were applied. Before the experiment started, participants were able to individually calibrate US volume between 90 and 95 dB (Bierwirth et al. 2021; Lonsdorf et al. 2017; Sperl et al. 2016) so that the noise was highly uncomfortable but not painful (Mueller et al. 2014). Afterwards, the experiment started with a habituation phase, consisting of five presentations of the US and subsequently four presentations of each CS. US habituation trials were performed to allow motor startle responses to stabilize (Abel et al. 1998; Blumenthal 1997; Winslow et al. 2002). Then, the acquisition phase started with the pseudo‐randomized presentation of 20 unfiltered presentations of each CS. Here, two out of four identities were intermittently paired with the US in 50% of occurrences (CS+) while the remaining two were not paired with any sound (CS−). Partial reinforcement was chosen to delay extinction in the decisive retrieval phase described below and to ensure a sufficient number of CS trials without subsequent US presentations for the analysis of the acquisition phase (Lonsdorf et al. 2017). The assignment of face identities to the CS conditions was randomized across participants. All CS were presented for 2000 ms, the US was presented 1000 ms after CS+ onset. The interstimulus interval consisted of a white fixation cross with a variable presentation time of 3000–3500 ms to reduce stimulus onset predictability. Overall, the first acquisition phase consisted of 80 trials. In the ensuing retrieval phase, CS were presented in either LSF or HSF. The presentation duration was randomly varied so that approximately 50% of stimuli in each CS × SF condition were presented for 100 ms, and 50% for 1000 ms. Each CS × SF × Presentation time cell consisted of 20 trials, leading to a total of 160 trials per retrieval block. To avoid early extinction in the retrieval phase (Bierwirth et al. 2023; Lissek et al. 2008; Sperl et al. 2019), CS+ were still randomly paired with a US after 200 ms with a 25% chance. An exemplary trial run is illustrated in Figure 1b.

Acquisition and retrieval blocks were repeated two more times. The acquisition blocks were reduced to 40 trials per block (10 trials/identity). Trial count in the retrieval block was equal across blocks. Blocks were separated by self‐paced breaks. Across all phases, consecutive presentations of the same identity were constrained to a maximum of four trials. After the last retrieval phase, we included a brief test phase, presenting all CS identities and no US for four times each (16 trials in total). Afterwards, participants were asked to rate both arousal and valence of all four CS identities on a 7‐point Likert scale (Bradley and Lang 1994). Furthermore, we assessed contingency awareness and US expectancy during the retrieval phase with questionnaires after the experiment ended. Contingency awareness was considered given if participants correctly indicated the regularity in the occurrence of the US (“Did the occurrence of the sound follow a specific pattern?”) or explicitly described the CS+ faces as aversive and CS− faces as neutral/non‐aversive (“Which association do you have with this face”). Thirty‐three participants (49.28%) correctly identified the CS‐US contingencies, while 35 participants did not identify a pattern in the CS‐US pairings. For US expectancy, 44.93% of participants reported that they expected the US for up to 10 trials in the retrieval phase, 40.58% expected it for up to 20 trials, and 11.59% expected it for more than 20 trials. In total, the experiment consisted of 672 CS presentations and took about 60 min.

### 2.4 Dependent Measures

#### 2.4.1 Ratings

Ratings of arousal and valence were given for all four CS identities on a 7‐point Likert scale (Bradley and Lang 1994). For valence, higher scores (minimum = 1, maximum = 7) indicated a more positive valence. For arousal, higher scores (minimum = 1, maximum = 7) indicated higher subjective arousal. No information regarding the US contingency was given when ratings were applied.

#### 2.4.2 EEG

The EEG was continuously recorded from 32 active BioSemi Ag‐AgCl electrodes using the ActiveTwo system and ActiView software (http://www.biosemi.com/). The recording was referenced to the Cz electrode, with the sampling rate set to 1024 Hz. Following BioSemi recommendations, electrode offsets were kept below ±25 mV during recording. NaCl contact electrolyte gel was used to improve surface conductivity. Pre‐processing of offline data was performed using the FieldTrip toolbox (Version 20240414, Oostenveld et al. 2011) in MATLAB (Version 2019b, The MathWorks Inc.). Continuous offline data were re‐referenced to the common average of all EEG channels. A first‐order zero‐phase filter high‐pass Butterworth of 0.1 Hz and a first‐order zero‐phase low‐pass Butterworth filter of 40 Hz were applied to ensure accurate detection of sharp peak components like the P1 (Widmann et al. 2015). Filtered data were cut into segments of −2000 to 2000 ms relative to stimulus onset and subsequently down‐sampled to 250 Hz. Baseline‐correction used 200 ms before stimulus onset. Bad channels were identified using a *z*‐value based threshold and temporarily removed before independent component analysis (ICA). Datasets with > 10 bad channels were excluded from the analysis. ICA was performed to identify and remove components associated with ocular, muscle, and technical artifacts. On average, 11.23% of channels were interpolated per participant. After the ICA, previously excluded channels were interpolated using a weighted neighboring approach. On average, 4.55% of channels were interpolated. Finally, if single trials exceeded a maximum amplitude range of 250 μV, they were excluded. For statistical analysis, data were averaged across time‐points and channel clusters of the P1 and N1. In line with previous studies on facial threat differentiation in the P1 (Müller‐Bardorff et al. 2018; Schindler, Bruchmann, et al. 2021; Weidner et al. 2025), we scored the component at two symmetrical parieto‐occipital electrode clusters (P3, PO3, O1 and P4, PO4, O2, Figure 1d) from 90 to 130 ms after stimulus onset. Although channels P7 and P8 were pre‐registered as part of the channel cluster of interest, they were excluded from the analysis because they fell outside the topographic distribution of the P1 (see Figure 1d). For the N1, we scored the component at two symmetrical parietal electrodes (P7 and P8, Figure 1d) from 140 to 190 ms after stimulus onset during the habituation and acquisition phase. During the retrieval phase, the N1 was scored from 150 to 210 ms as its peak latency was delayed. The delay may have occurred due to the spatial filtering of the faces (Pourtois, Dan, et al. 2005). Please note that due to the lateral topographic distribution of the N1, the analyzed channel cluster deviates from the pre‐registered cluster (see Figure 1).

#### 2.4.3 SCR

For the analysis of the SCR, recommendations by Boucsein (2012) were followed. Electrodermal activity was recorded with two Ag‐AgCl electrodes placed on the thenar and hypothenar of the non‐dominant hand. Skin was pretreated with 70% ethanol. NaCl contact electrolyte gel was used to improve surface conductivity. Data were recorded with the BioSemi EEG amplifier. The sampling rate was 1024 Hz. Offline data were processed with custom scripts in MATLAB and the Fieldtrip toolbox. Continuous offline data were resampled to 10 Hz. A high‐pass Butterworth first‐order zero‐phase filter of 0.1 Hz and a low‐pass first‐order zero‐phase Butterworth filter of 10 Hz were applied. Phasic skin conductivity was extracted in response to each stimulus −500 to 6000 ms after stimulus onset. SCR were manually scored as the trough‐to‐peak difference by using a custom‐made program according to published guidelines (Boucsein 2012) and blind to stimulus type. Peaks were defined relative to the response onset. Only the first SCR response per trial was scored, with the scoring window truncated at the onset of any overlapping response. SCR were required to reach a minimum amplitude of 0.02 mS (Boucsein 2012; Kuhn et al. 2022). The trough was identified in an onset latency window of 900–4500 ms post‐stimulus onset and the first peak was identified in a peak detection window (PDW) of maximally 6000 ms post‐stimulus onset. This corresponds to the so‐called “first‐interval response” (Kuhn et al. 2022).

To address potential carryover effects of SCR onsets to the next trial, arising from the short inter‐stimulus interval (ISI) in the retrieval phase, we calculated the proportion of trials in which the SCR onset occurred after the onset of the subsequent stimulus (100 ms stimulus duration + average of 3250 ms ISI = 3350 ms until the next stimulus onset). For the 100 ms retrieval condition, 22.06% (SD = 5.33) of trials showed a response onset beyond this window. Given this substantial number of trials where carryover effects could have occurred, we conducted additional analyses excluding these trials and re‐running the analyses (see Section 2.5 for model specification) for SCR without the affected trials. The pattern of results did not change after exclusion of the carryover trials (see Table S10).

CS+ trials with subsequent US presentations were excluded to avoid overlapping responses in the SCR (Lonsdorf et al. 2017). To ensure comparability between EEG and SCR data, we used the same approach for the EEG analysis. To even out trial counts between CS+ and CS−, we randomly excluded CS− trials before statistical analysis. No additional trial rejection was done for the SCR, but participants classified as hyper‐responders (≥ 95% of US response amplitudes, *n* = 3) or non‐responders (no US responses, *n* = 2) were excluded from the SCR analysis (Lonsdorf et al. 2019). The number of remaining trials in the SCR analysis was M = 39.685 (SD = 0.627) trials per condition in the acquisition phase and M = 44.728 (SD = 4.964) trials per condition in the retrieval phase. Trial counts did not differ between SF or duration conditions (SF: *F*(1, 33) = 1.845, *p* = 0.184; duration: *F*(1, 33) = 0.463, *p* = 0.501).

Because of the additional artifact correction, the number of remaining trials in the EEG analysis differed slightly from the SCR analysis. The number of remaining trials was M = 39.236 (SD = 2.765) trials per condition in the acquisition phase and M = 43.272 (SD = 5.601) trials per condition in the retrieval phase. Additionally, trial counts did not differ between SF or duration conditions (SF: *F*(1, 67) = 0.467, *p* = 0.497; duration: *F*(1, 67) = 0.476, *p* = 0.493).

### 2.5 Statistical Analysis

#### 2.5.1 General Approach

All statistical analyses were conducted in R (version 2023.03.1, R Core Team 2021) using dummy‐coded hierarchical linear mixed models (LMM, *lme4* v.1.1‐34; Figure 1d). Unstandardized regression coefficients are reported as effect sizes. This method allows direct inferences about the direction of a given effect and interaction due to a priori defined contrasts (Frömer et al. 2018). Significance of fixed‐effects Satterthwaite *t*‐values was evaluated with non‐parametric Monte Carlo permutation tests using a maximum summation approach (Maris and Oostenveld 2007; for a similar approach, see Weidner et al. 2025). Specifically, within‐subject labels were permuted 1000 times, and for each permutation step, a new LMM was fitted. The distribution of permuted *t*‐values was then used to compute empirical *p*‐values, defined as the proportion of permuted *t*‐values greater than or equal to the observed statistic. All tests were two‐tailed. Post hoc comparisons were performed with the predicted marginal means using *emmeans* (v.1.8.8, Lenth 2018). With Holm corrections of post hoc tests, we accounted for the inflation of false positives in multiple testing (Holm 1979). For the post hoc tests, Cohen's *d* is reported as the effect size. It is interpreted as small when *d* = 0.20, medium when *d* = 0.50, and large when *d* = 0.80 (Cohen 1988, 1992). All data were analyzed based on single‐trial data, with subject included as random intercepts to account for repeated measures. Post hoc power estimations of LMM coefficients were conducted with the *simr* package running with 500 simulations (Green and MacLeod 2016). Post hoc power was not estimated for null‐effects and exploratory analyses. If not stated otherwise, only significant effects are reported in the text. For readability, *t*‐values and degrees of freedom derived from LMMs are not reported in the main text but in the corresponding Supplementary Tables.

#### 2.5.2 Analysis Models

For the subjective ratings of valence and arousal, ratings per identity were assigned to the CS conditions. Separate models were fit for the two rating measures. Rating analyses were carried out based on the EEG sample including participants without SCR data (*n* = 68). For electrophysiological responses, we tested whether responses differed between broadband filtered CS+ and CS− during the habituation and acquisition phase. These comparisons were based on a one‐factorial design, with P1 amplitudes and phasic SCR peaks analyzed as a function of CS (CS+ vs. CS−, reference level: CS−). Since face processing is typically right‐lateralized (Kanwisher et al. 1997; Rossion et al. 2003), a second factor hemisphere was introduced for the P1 and N1 analysis (left vs. right, reference level: right) to test for potential lateralization effects.

For the decisive analyses of the retrieval phase, we employed a 2 × 2 repeated‐measures design with the fixed‐effects CS (CS+ vs. CS−, reference level: CS−) and SF (LSF vs. HSF, reference level: HSF). Again, the third factor hemisphere (left vs. right, reference level: right) was additionally introduced for the P1 and N1 analyses. Furthermore, because the long‐latency SCR might be sensitive to the manipulation of stimulus duration, we added the fixed‐effect duration (100 ms vs. 1000 ms) in the SCR LMM.

#### 2.5.3 P1‐SCR Association

To test for the association of P1 and SCR data during the retrieval phase, we calculated an additional LMM, predicting trial‐wise SCR values based on P1 amplitudes in interaction with the fixed‐effects CS, SF, and hemisphere. Before analysis, P1 and SCR data were *z*‐transformed. For post hoc tests of regression slopes, unstandardized beta coefficients are reported as effect sizes in addition to the Std‐*β*. Only trials that were present both in the P1 and SCR analysis were considered for analysis, and the number of trials was balanced between CS conditions (see above). The LMM was specified with the additive fixed‐effect trial to avoid inflating the correlation of SCR and P1 amplitudes by potentially simultaneous signal habituation (Lonsdorf et al. 2017). Random slopes were added to the model to let slopes vary per participant.

## 3 Results

### 3.1 Stimulus Ratings

Figure 1c illustrates the rating results of all participants. Both rating scales differentiated threat (CS+) from safety (CS−) faces, with lower valence (*β* = −0.804, *p* = 7.12e^−06^. Power (M[CI]) = 99.60 (98.56, 99.95) %) and higher arousal scores for CS+ than CS− (*β* = 0.449, *p* = 0.004, Power (M[CI]) = 85.40 (82.00, 88.38) %). All model coefficients are documented in Table S1.

### 3.2 P1

#### 3.2.1 Habituation: Broadband Presentation

The LMM indicated a significant fixed‐effect hemisphere during the habituation phase, with higher P1 amplitudes over the left than right hemisphere (*β* = −0.933, *p* = 0.002; Power (M[CI]) = 96.40 (94.37, 97.85) %). No significant effect of CS or a CS × hemisphere interaction was observed. Separate P1 analyses with the subsample of participants who also had available SCR data confirmed the same pattern of activity found in the full sample, although the main effect of hemisphere reached only marginal significance (*p* = 0.067). All model coefficients are documented in Table S2.

#### 3.2.2 Acquisition: Broadband Presentation

The LMM indicated a significant fixed effect of hemisphere during the acquisition phase, with higher P1 amplitudes over the left than right hemisphere (*β* = −0.314, *p* = 0.028; Power (M[CI]) = 78.40 (74.53, 81.93) %). No significant effect of CS or a CS × hemisphere interaction was observed. Separate P1 analyses with the subsample of participants who also had available SCR data confirmed the same pattern of activity as found in the full sample. All model coefficients are documented in Table S3.

#### 3.2.3 Retrieval: Filtered Presentation

The LMM revealed significant differences between CS+ and CS− in the P1 amplitude during the retrieval phase (CS+ > CS−; *β* = 0.205, *p* = 0.029, Power (M[CI]) = 86.40 (83.08, 89.28) %). Furthermore, as seen in Figure 2, the three‐way interaction CS × SF × hemisphere was significant (*β* = 0.409, *p* = 0.029, Power (M[CI]) = 60.20 (55.76, 64.52) %). Post hoc tests revealed that this was driven by lateralized changes in CS‐differentiation based on spatial frequency: the largest CS‐differentiation was found for LSF faces over the left hemisphere (*β* = −0.268, *p* = 0.012, *d* = −0.326). Over the right hemisphere, marginally larger P1 amplitudes for CS+ versus CS− faces were found for HSF faces (*β* = −0.205, *p* = 0.058, *d* = −0.244). No significant CS‐differences were observed for LSF over the right or for HSF over the left hemisphere. Separate P1 analyses with the subsample of participants who also had available SCR data confirmed the same pattern of activity as found in the full sample. All model coefficients are documented in Table 1, post hoc tests are found in Table S4.

**FIGURE 2:**
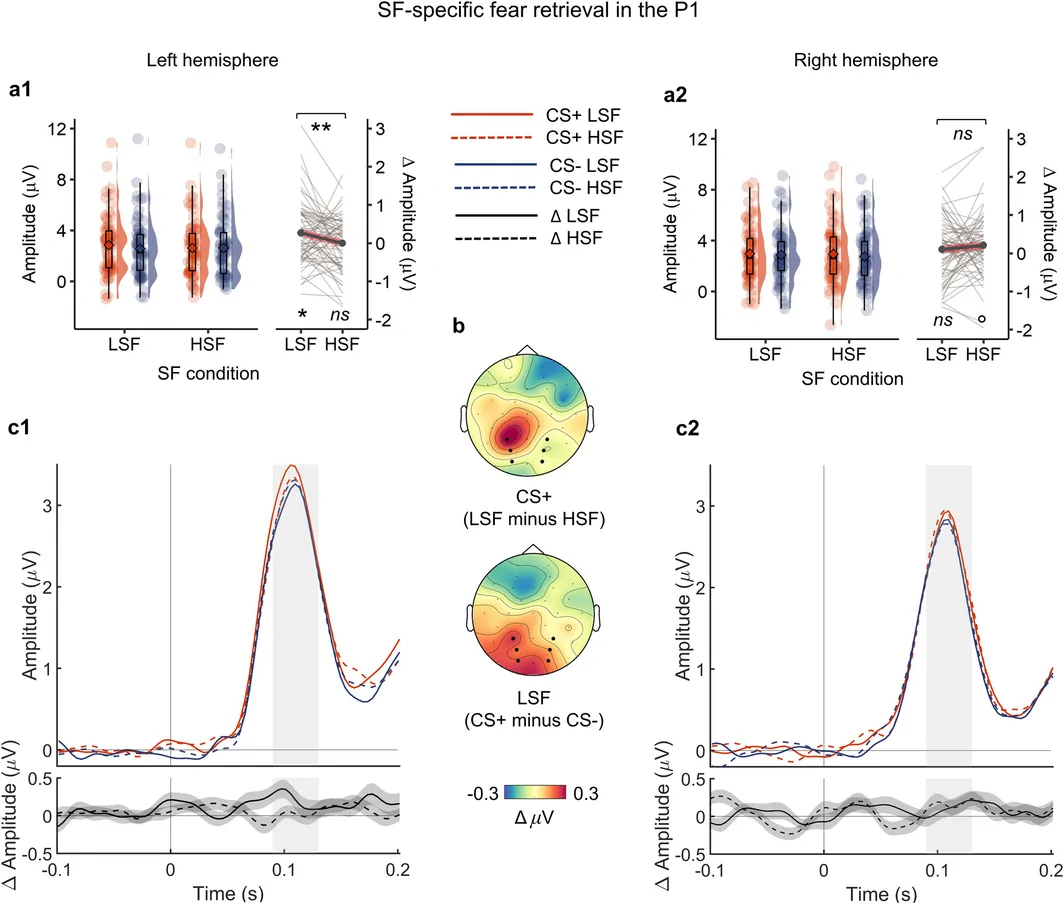
CS‐differentiation in P1 amplitude during the retrieval phase. (a1/a2) Distribution of P1 amplitudes in response to each CS × SF condition for the left (a1) and right (a2) hemisphere. Boxplots show median and interquartile range; raincloud plots display single‐subject means per CS × SF condition along with the smoothed distribution density; outlined diamonds indicate the overall condition mean. Line plots depict the CS‐differentiation (CS+ minus CS−) within SF conditions. Bold gray lines indicate mean group differences; red ribbons indicate standard error of the difference; light gray lines depict single‐subject mean differences. Stars and brackets mark significant post hoc tests (°*p* ≤ 0.10, *p ≤ 0.05, **p ≤ 0.01). (b) Differential (top: CS+ LSF vs. CS+ HSF, bottom: CS+ LSF vs. CS‐LSF) scalp topography during the P1‐time window (90–130 ms). Channels that were used to extract the P1 are marked in black. (c1/c2) Average (top panel) and differential (CS+ vs. CS−, bottom panel) evoked time‐series of responses in the left (c1) and right (c2) channel cluster. The shaded area marks the P1 time‐window. CS, conditioned stimulus; LSF, low spatial frequency; HSF, high spatial frequency; ns, not significant; SF, spatial frequency.

**TABLE 1:** P1 retrieval: linear mixed model fixed effects.

Term	Estimate	SE	df	*t*	*p*
Full sample					
(Intercept)	**2.743**	**0.257**	**75.482**	**10.665**	**1.01e** ^**−16**^
CS	**0.205**	**0.093**	**47,005.211**	**2.189**	**0.029**
SF	0.145	0.094	47,005.393	1.554	0.120
Hemisphere	−0.121	0.093	47,005.269	−1.296	0.195
CS × SF	−0.131	0.132	47,005.325	−0.993	0.321
CS × Hemisphere	−0.214	0.132	47,005.138	−1.620	0.105
SF × Hemisphere	−0.201	0.132	47,005.271	−1.517	0.129
CS × SF × Hemisphere	**0.409**	**0.187**	**47,005.139**	**2.185**	**0.029**
SCR subsample					
(Intercept)	**2.795**	**0.393**	**38.463**	**7.113**	**1.62e** ^**−08**^
CS	0.207	0.128	24,509.103	1.626	0.104
SF	0.143	0.128	24,509.215	1.118	0.254
Hemisphere	0.022	0.127	24,509.122	0.174	0.862
CS × SF	−0.162	0.180	24,509.179	−0.900	0.355
CS × Hemisphere	−0.351	0.180	24,509.065	−1.951	0.057
SF × Hemisphere	−0.263	0.180	24,509.146	−1.459	0.158
CS × SF × Hemisphere	**0.566**	**0.254**	**24,509.078**	**2.223**	**0.031**

*Note:* *T*‐statistics are evaluated for significance. A positive coefficient indicates higher values in the test condition than in the reference group. Effects with *p* ≤ 0.05 are marked in bold.

Abbreviations: CS, conditioned stimulus; SCR, skin conductance response; SE, standard error; SF, spatial frequency.

### 3.3 N1

#### 3.3.1 Habituation: Broadband Presentation

The LMM indicated no significant fixed effects in the N1 during the habituation phase. Separate N1 analyses with the subsample of participants who also had available SCR data confirmed the same pattern of activity found in the full sample. All model coefficients are documented in Table S5.

#### 3.3.2 Acquisition: Broadband Presentation

As seen in Figure 3, the LMM indicated a marginally significant fixed effect of CS during the acquisition phase, with larger (more negative) N1 amplitudes for CS+ than CS− faces (*β* = −0.278, *p* = 0.070; Power (M[CI]) = 52.60 (48.12, 57.05) %). Separate N1 analyses with the subsample of participants who also had available SCR data confirmed the same pattern of activity as found in the full sample. All model coefficients are documented in Table 2.

**FIGURE 3:**
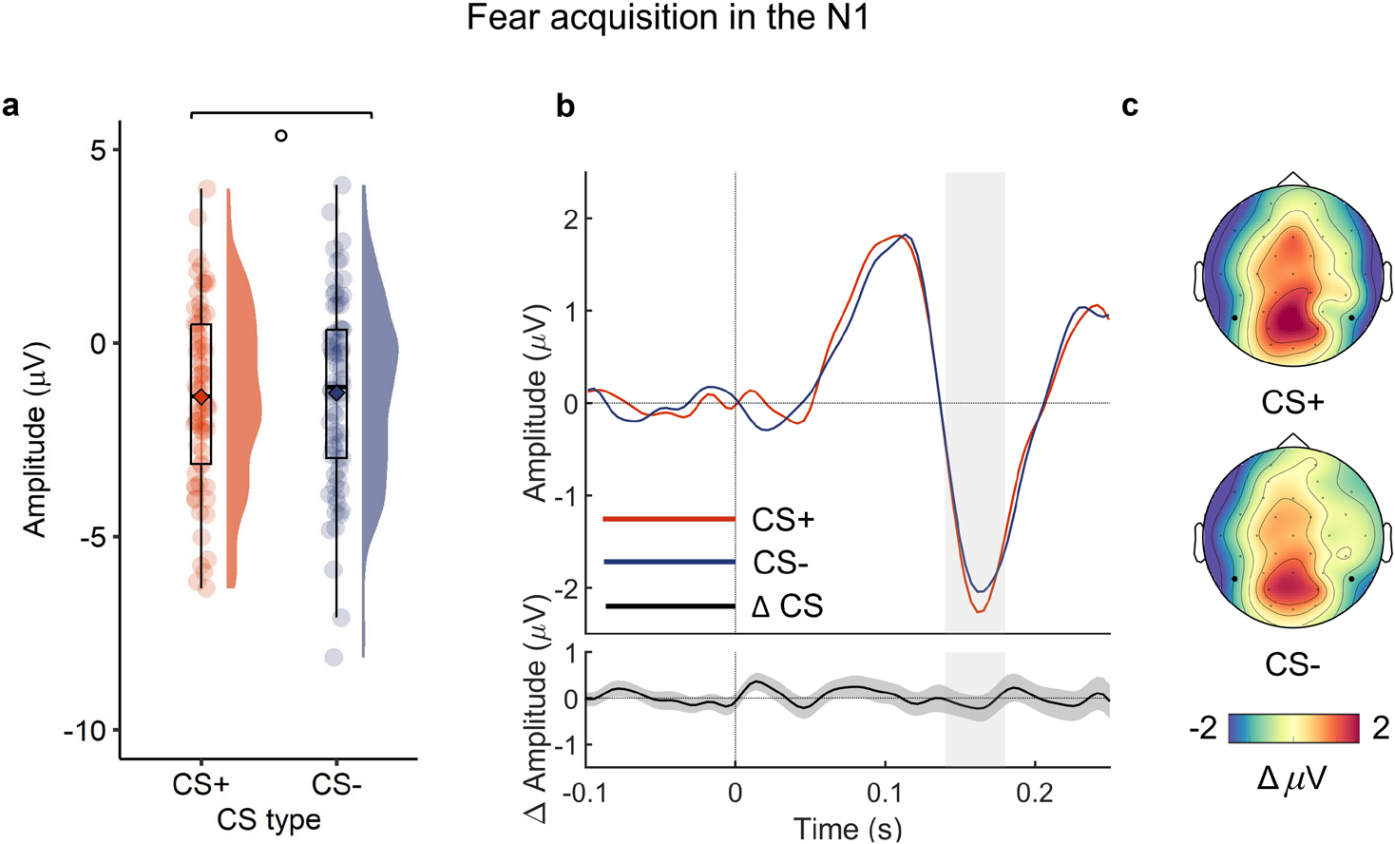
CS‐differentiation in N1 amplitude during the acquisition phase. (a) Distribution of N1 amplitudes in response to each CS condition, averaged across both hemispheres. Boxplots show median and interquartile range; raincloud plots display single‐subject means per CS condition along with the smoothed distribution density; outlined diamonds indicate the overall condition mean. Stars and brackets mark marginally significant post hoc tests (°*p* ≤ 0.10). (b) Average (top panel) and differential (CS+ vs. CS−, bottom panel) evoked time‐series of responses in N1 channel cluster. The shaded area marks the N1 time‐window. (c) Scalp topographies of the CS conditions during the N1‐time window (140–190 ms). Channels that were used to extract the N1 are marked in black. CS, conditioned stimulus.

**TABLE 2:** N1 acquisition: linear mixed model fixed effects.

Term	Estimate	SE	df	*t*	*p*
Full sample					
(Intercept)	**−1.169**	**0.306**	**89.609**	**−3.818**	**2.47e** ^**−04**^
CS	−0.278	0.151	15,819.185	−1.834	0.070
Hemipshere	−0.224	0.174	15,819.046	−1.286	0.209
CS × Hemipshere	0.330	0.214	15,819.046	1.541	0.128
SCR subsample					
(Intercept)	**−2.249**	**0.436**	**39.061**	**−5.158**	**7.56e** ^**−06**^
CS	−0.381	0.212	8347.091	−1.797	0.070
Hemipshere	**−0.601**	**0.174**	**8347.012**	**−3.452**	**5.59e** ^**−04**^
CS × Hemipshere	0.290	0.300	8347.012	0.968	0.340

*Note:* *T*‐statistics are evaluated for significance. A positive coefficient indicates higher values in the test condition than in the reference group. Effects with *p* ≤ 0.05 are marked in bold.

Abbreviations: CS, conditioned stimulus; SCR, skin conductance response; SE, standard error.

#### 3.3.3 Retrieval: Filtered Presentation

The LMM revealed significant differences between LSF and HSF faces in the N1 amplitude during the retrieval phase (LSF < HSF; *β* = −0.713, *p* = 3.58e^−08^, Power (M[CI]) = 100 (99.26, 100) %, Figure S1). No other significant effects were observed. Separate N1 analyses with the subsample of participants who also had available SCR data confirmed the same pattern of activity as found in the full sample. All model coefficients are documented in Table S6.

### 3.4 SCR

#### 3.4.1 Habituation: Broadband Presentation

Investigating the SCR during the habituation phase, the LMM revealed no significant effect of CS (*β* = 0.021, *p* = 1). All model coefficients are documented in Table S7.

#### 3.4.2 Acquisition: Broadband Presentation

Investigating the SCR during the acquisition phase, the LMM revealed no significant effect of CS (*β* = 0.00003, *p* = 0.930). All model coefficients are documented in Table S8.

#### 3.4.3 Retrieval: Filtered Presentation

As seen in Figure 4a, the LMM revealed a significant CS × duration interaction (*β* = 0.017, *p* = 0.021; Power (M[CI]) = 46.60 (42.16, 51.08) %). CS‐differentiation increased for longer (1000 ms) stimulus durations, although this reached only marginal significance in the Holm‐corrected post hoc tests (1000 ms: CS− vs. CS+: *β* = −0.007, *p* = 0.071, *d* = −0.179; 100 ms: CS− vs. CS+: *β* = 0.005, *p* = 0.218, *d* = 0.259). The model also revealed a significant effect of duration (100 ms > 1000 ms; *β* = −0.012, *p* = 0.022; Power (M[CI]) = 48.80 (44.34, 53.28) %) as well as a significant SF × duration interaction (*β* = 0.016, *p* = 0.035; Power (M[CI]) = 51.60 (47.12, 56.60) %). Post hoc tests showed that SCR amplitudes marginally increased in response to LSF versus HSF faces when shown for 1000 ms (1000 ms: LSF > HSF: *β* = −0.007, *p* = 0.072, *d* = −0.255; 100 ms: LSF = HSF: *β* = 0.003, *p* = 0.342, *d* = 0.182). All model coefficients are documented in Table 3, post hoc tests are found in Table S9. SCR during the retrieval phase were largely identical after accounting for potential response carryovers in the 100 ms duration condition, although the previously significant CS × duration interaction was now reduced to marginal significance (*p* = 0.096) (see Table S10).

**FIGURE 4:**
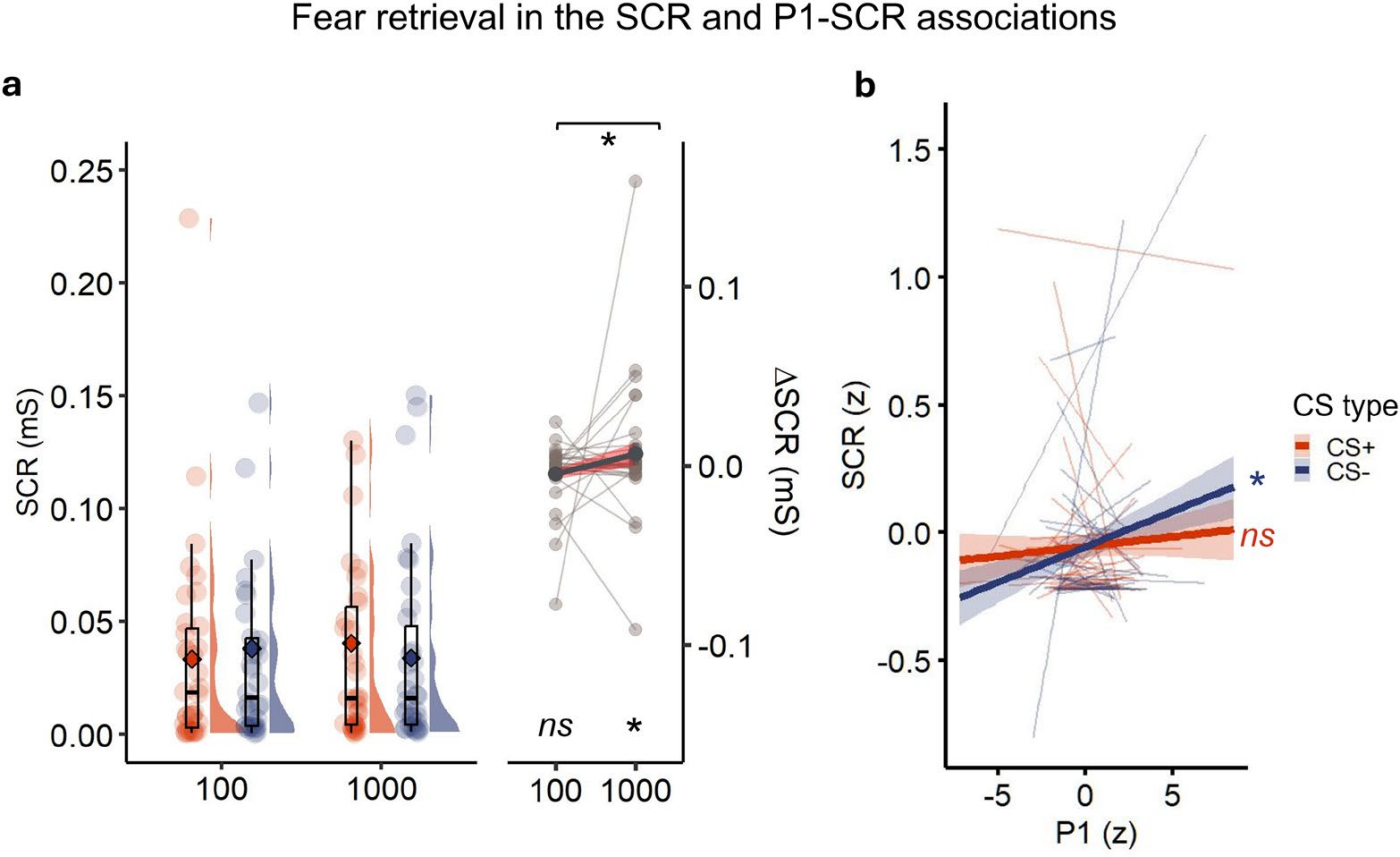
CS‐differentiation in SCR and association with P1 amplitudes. (a) SCR amplitude in response to the CS × duration conditions. Boxplots show median and interquartile range; raincloud plots display single‐subject means per CS × duration condition along with the smoothed distribution density; outlined diamonds indicate the overall condition mean. Line plots depict the CS‐differentiation (CS+ vs. CS−) within duration conditions. The bold gray line indicates mean group differences; red ribbons indicate standard error of the difference; light gray lines depict single‐subject mean differences. (b) *Z*‐transformed P1‐SCR associations within the CS conditions. Opaque line plots depict standardized linear regression slopes of SCR amplitude based on P1 amplitude along with the standard error (shaded area). These slopes reflect trial‐by‐trial P1‐SCR relationships with random slopes and intercepts per subject. Transparent lines display single‐subject slopes per CS condition. Stars and brackets mark significant post hoc tests (*p ≤ 0.05). CS, conditioned stimulus; ns, not significant; SCR, skin conductance response.

**TABLE 3:** SCR retrieval: linear mixed model fixed effects.

Term	Estimate	SE	df	*t*	*p*
Full sample					
(Intercept)	**0.042**	**0.009**	**44.23**	**4.576**	**0.002**
CS	−0.010	0.005	12,125.80	−1.847	0.069
SF	−0.009	0.005	12,125.60	−1.672	0.090
Duration	**−0.012**	**0.005**	**12,125.86**	**−2.282**	**0.022**
CS × SF	0.010	0.007	12,125.78	1.388	0.167
CS × Duration	**0.017**	**0.007**	**12,126.38**	**2.256**	**0.021**
SF × Duration	**0.016**	**0.007**	**12,126.24**	**2.118**	**0.035**
CS × SF × Duration	−0.011	0.010	12,126.21	−1.053	0.303

*Note:* *T*‐statistics are evaluated for significance. A positive coefficient indicates higher values in the test condition than in the reference group. Effects with *p* ≤ 0.05 are marked in bold.

Abbreviations: CS, conditioned stimulus; SCR, skin conductance response; SE, standard error; SF, spatial frequency.

### 3.5 P1‐SCR Association

The model revealed a significant prediction of SCR amplitudes by P1 amplitudes that varied based on the CS condition (EEG × CS: *β* = 0.211, *p* = 0.047, Figure 4b). Post hoc tests revealed that the only significant association of SCR amplitudes with P1 amplitudes was present for CS− faces (CS−: Std‐*β* = 0.028, *β* = 0.0007, *p* = 0.039; CS+: Std‐*β* = 0.008, *β* = 0.0004, *p* = 0.562). All model coefficients are documented in Table S11; post hoc tests are found in Table S12.

## 4 Discussion

Previous studies suggest that detection of facial threat in early visual attention, as reflected in the P1 component, may be driven by LSF visual information while SF‐sensitivity of fear differentiation in face encoding in the N1 has been debated. Crucially, it remained unclear whether any such effects, often investigated by comparing responses to fearful versus neutral faces, are due to inherent differences between expressions or genuine detection of threat relevance (Bruchmann et al. 2020; Schindler, Bruchmann, et al. 2021; Schindler, Wolf, et al. 2021; Yu et al. 2023). To address these issues, we investigated the contribution of specific spatial frequencies to P1 and N1 threat detection in newly acquired fear associations of faces with neutral expressions. Furthermore, we explored whether different visual pathways of fear processing are reflected in SCR, and whether shared pathways of fear signals contribute to P1 and SCR amplitudes.

First, we could demonstrate that threat detection in the P1 component is retained even in the absence of inherent physical cues of threat‐specific facial expressions. This contradicts previous hypotheses suggesting that mainly perceptual factors underlie P1 sensitivity to threat‐related faces (Schindler, Wolf, et al. 2021), although they might play a modulatory role. Indeed, confirming our pre‐registered hypotheses, P1 fear retrieval was distinctly shaped by the SF‐content of the face, albeit in a lateralized manner: P1 amplitudes showed largest amplitudes in response to LSF CS+ faces over the left hemisphere during fear retrieval, whereas over the right hemisphere, threat detection was, in tendency, mediated by HSF. Habituation analyses confirmed no pre‐conditioning differences in CS responses. Ratings and a trend in N1 amplitudes confirmed successful fear acquisition while no conditioning effects during the acquisition phase were observed in the P1 and SCR. Potentially, too few trials with established US‐contingencies were available for robust acquisition effects (Bach et al. 2023). This might have been due to the partial reinforcement rate that can lead to slower contingency learning (Grady et al. 2016). Against our hypotheses, SCR sensitivity towards CS+ faces was not driven by SF but by long stimulus durations. P1 and SCR conditioning effects were positively correlated only for CS− faces.

P1 data from the retrieval phase support the assumption that magnocellular signals can drive the retrieval of threat associations during early stages of visuocortical attention (Carretié et al. 2017; Méndez‐Bértolo et al. 2016; Morris et al. 1999), even when faces have a neutral expression. Our results highlight the ability of magnocellular‐cortical pathways to adapt remarkably quickly (Cinca‐Tomás et al. 2025; Weinberger 1982; Zhuang et al. 2015) and suggest that magnocellular learning might underlie the initial formation of threat associations. Notably, CS‐differentiation of LSF faces effect was constrained to the left hemisphere. This contrasts with the well‐known right‐hemispheric dominance in face processing (Haxby et al. 2000; Kanwisher et al. 1997; Kanwisher and Yovel 2006) and suggests additional left lateralized mechanisms of threat perception that are specific to LSF information. Indeed, particularly visual discrimination in the left hemisphere has been linked to LSF processing (Kitterle et al. 1990; Peyrin et al. 2003) and magnocellular signaling (Okubo and Nicholls 2005). More specifically, the presently observed temporo‐parietal focus of SF differences for CS+ faces could point towards left‐hemisphere dominant magnocellular projections to the dorsal visual stream (Bullier 2001; Maunsell et al. 1990; Nassi and Callaway 2006; Skottun 2015; Tootell and Nasr 2017; You and Li 2016). According to Carretié et al. (2020), it is likely that threat information is directly evaluated within magnocellular subdivisions projecting to the dorsal visual stream and entrained by the amygdala (Cinca‐Tomás et al. 2025; Maren et al. 2001). As the dorsal visual stream is implicated in attention and spatial orientation (Corbetta et al. 2008; Goodale and Milner 1992; Milner and Goodale 2006), its connections to the magnocellular divisions of the thalamus might support rapid, attentional engagement with the fear‐associated stimulus (Pourtois, Thut, et al. 2005). Indeed, saccadic activity was observed to be faster for threatening than neutral images when preceded by an LSF prime (Zhu et al. 2021).

In tendency, right‐hemispheric P1 amplitudes showed CS‐differentiation for HSF faces. As a speculative explanation, this trend may imply a stronger reliance on parvocellular threat signals in right‐hemispheric ventral visual areas (Li and Keil 2023; Steinberg et al. 2012). As part of the right‐dominant face processing network (Fox et al. 2009; Kanwisher et al. 1997; Kanwisher and Yovel 2006), these regions are implicated in structural encoding of faces (de Cesarei et al. 2013; Schindler, Bruchmann, et al. 2021) and may rely on detailed HSF information (Rebaï et al. 1998; You and Li 2016; Zhao et al. 2023). Therefore, it appears that multiple, parallel pathways of early visual threat processing adapt to distinct visual features during learning (Bruchmann et al. 2023; Denison and Silver 2012).

In accordance with the present CS‐differentiation in the N1 during fear acquisition, the N1 has been linked to sensory learning (Olofsson et al. 2008; Ugland et al. 2013). The CS‐differentiation was marginal; however, potentially because fear associations have not been fully established (Grady et al. 2016). During retrieval, the absence of N1 CS‐differentiation might indicate a loss of facial feature information (Schindler et al. 2022) or contrast (Han et al. 2003) in the filtered faces precluding associative responses in the N1 (Pourtois, Dan, et al. 2005). Face encoding in the N1 is thought to rely on spatial frequencies of roughly 8–16 cycles/image (Halit et al. 2006), which falls almost completely outside of the range used in the present study but might explain why LSF faces resulted in generally larger N1 amplitudes than HSF faces.

In the SCR, higher autonomic arousal in response to CS+ than CS− faces during the retrieval phase was observed only for faces that were presented for longer durations. Increased time to engage with the stimulus might have facilitated fear retrieval in the SCR because it allows for more detailed categorization of facial identity (Tanskanen et al. 2007) which was the key‐predictor of US‐contingency in the present study. Correspondingly, greater SCR sensitivity to CS+ than CS− was linked to associative processing in frontal areas (Andreatta et al. 2015; Carter et al. 2006; McIntosh et al. 1999; Mechias et al. 2010). In parallel, longer presentation times were also associated with higher SCR to LSF than HSF faces. Park et al. (2016) argue that, due to the assumed involvement of the amygdala in the magnocellular pathway, particularly LSF stimuli may elicit higher arousal. Correspondingly, the categorization of LSF identities was previously shown to be skewed towards negative interpretations (Neta and Whalen 2010; Park et al. 2016). Hence, against our hypotheses, the present findings suggest that the SCR reflects conscious anticipatory arousal (Baeuchl et al. 2019; Sevenster et al. 2014) rather than SF‐specific fear differentiation. These may be more reliably present during unconscious perception of threat‐stimuli (Chen et al. 2023), which was not part of our study. Similarly, correlation analyses suggested that P1 and SCR fear differentiation are not driven by shared magnocellular input but rather shared input from instances encoding the CS−, for example, the hippocampus and frontal areas (Lissek et al. 2014), that are relevant for the inhibition of threat overgeneralization (Kaczkurkin et al. 2017).

Despite these novel insights into SF‐specific threat retrieval, some limitations must be considered when interpreting the present findings. Firstly, the inclusion of the US during the habituation phase, while intended to minimize startle‐related artifacts, may have accelerated US habituation and potentially attenuated conditioned responding in subsequent phases. The relatively low number of contingency‐aware participants (Li et al. 2025) may reflect reduced US salience or US habituation across the experiment. Also, the carryover rate of SCR onsets exceeding the subsequent stimulus onset in the retrieval condition was about 20%, which was relatively high. Although the overall pattern of results remained largely unchanged after excluding these trials, the SCR data should be interpreted with caution. Future studies should implement longer inter‐stimulus intervals to ensure cleaner separation of physiological responses across trials. Finally, the present study did not focus on cortical responses earlier than the P1. Previous studies show fear‐conditioned effects to arise earlier in the C1 visual component (peaking approximately 40–80 ms post‐stimulus, Stolarova et al. 2006). Like the P1, the C1 was shown to be sensitive to SF characteristics of a visual stimulus (Foxe et al. 2008; Proverbio et al. 2010; Rebaï et al. 1998; Schmid et al. 2025). However, the present paradigm was not optimized for C1 detection as C1 amplitude and polarity are strongly determined by the retinal location of the stimulus relative to the calcarine sulcus (Clark et al. 1994). Hence, future studies might employ stimulus presentations targeting specific areas of the visual field to probe whether SF‐driven fear conditioning effects emerge as early as the C1.

In sum, this study provides compelling evidence that the magnocellular facilitation of visuocortical threat detection in faces goes beyond pre‐existing perceptual clues of specific facial expressions. It also indicates the presence of several parallel processes that differentially modulate early visual attention and autonomic arousal in response to potential threat. These findings refine models of emotional vision and demonstrate that particularly the P1 component can reflect rapid subcortical responses to threat associations. Understanding the neural circuits that underlie fear retrieval holds important implications for research on maladaptive fear responses found in anxiety (Chien et al. 2020; Kosteletou‐Kassotaki et al. 2026), or trauma‐related disorders (Careaga et al. 2016). Research should consider the different neural circuits of visual processing and their timing during fear retrieval. In doing so, it could potentially inform therapeutic protocols, such as neuromodulation techniques (Dittert et al. 2018; Zhang et al. 2024), exposure therapy (Lange et al. 2020; Winkler et al. 2025), or neurofeedback (Nicholson et al. 2017).

## Author Contributions

**Adrian Taday:** investigation, data curation. **Marlene Götze:** data curation, investigation, writing – review and editing. **Johanna Kissler:** supervision, resources, writing – review and editing. **Enya M. Weidner:** conceptualization, methodology, data curation, project administration, formal analysis, visualization, writing – original draft, writing – review and editing.

## Ethics Statement

The study was conducted in accordance with the Declaration of Helsinki and was approved by the Ethics committee of Bielefeld University (EUB‐2024_245), following the ethics guidelines of the German Psychological Association (DGPs).

## Conflicts of Interest

The authors declare no competing financial interests.

## Supporting information


**Table S1:** SAM ratings—linear mixed model fixed effects.
**Table S2:** P1 habituation: linear mixed model fixed effects.
**Table S3:** P1 acquisition: linear mixed model fixed effects.
**Table S4:** P1 retrieval: post hoc CS differences by SF × Hemisphere.
**Table S5:** N1 habituation: linear mixed model fixed effects.
**Table S6:** N1 retrieval: linear mixed model fixed effects.
**Table S7:** SCR habituation: linear mixed model fixed effects.
**Table S8:** SCR acquisition: linear mixed model fixed effects.
**Table S9:** SCR retrieval: post hoc CS/SF differences by duration.
**Table S10:** SCR—retrieval: linear mixed model fixed effects (without carryover trials).
**Table S11:** P1—SCR association: linear mixed model fixed effects.
**Table S12:** Post hoc tests P1—SCR association: linear mixed model fixed effects.
**Figure S1:** SF differentiation in N1 amplitudes during the retrieval phase. (a) Distribution of N1 amplitudes in response to each SF condition. Boxplots show median and interquartile range; raincloud plots display single‐subject means per SF condition along with the smoothed distribution density; outlined diamonds indicate the overall condition mean. Stars and brackets mark significant post hoc tests (***p ≤ 0.001). (b) Average (top panel) and differential (LSF vs. HSF, bottom panel) evoked time‐series of responses in the channel cluster. The shaded area marks the N1 time‐window. The topography depicts differential (LSF vs. HSF) scalp signals. Channels that were used to extract the N1 are marked in black. CS, conditioned stimulus; HSF, high spatial frequency; LSF, low spatial frequency; SF, spatial frequency.

## Data Availability

All data needed to evaluate the conclusions are present in the paper or the Supporting Information. Numerical source data for analyses, graphs, and charts are available under this link: https://gitlab.ub.uni‐bielefeld.de/ae02/weidner_subcortical_threat_retrieval. Raw data are available from the authors upon reasonable request to the corresponding author. Code Availability: The codes generated in this study are available under this link: https://gitlab.ub.uni‐bielefeld.de/ae02/weidner_subcortical_threat_retrieval.
