# Supplementary material for: Distinct Visual Pathways of Threat Retrieval in Fear‐Conditioned Faces
Source: Psychophysiology. 2026 Oct 14;63(10):e70429. doi: 10.1111/psyp.70429 (PMC13664660; doi:10.1111/psyp.70429)
Supplement: Supplementary file 1 [file PSYP-63-e70429-s001.docx]

# Supplementary Information for

Distinct visual pathways of threat retrieval in fear-conditioned faces

Enya M. Weidner^1*^, Marlene Götze^1^, Adrian Taday^1^, Johanna Kissler^1^

1 Department of Psychology, Bielefeld University, Universitätsstraße 25, 33615 Bielefeld, Germany

*Correspondence to: Enya Weidner

Email: [enya.weidner@uni-bielefeld.de](mailto:enya.weidner@uni-bielefeld.de)

# SAM ratings

**Table S1. SAM Ratings – Linear Mixed Model Fixed Effects**

| Scale | Term | Estimate | *SE* | *df* | *t* | *p* |
| --- | --- | --- | --- | --- | --- | --- |
| Arousal | (Intercept) | **4.297** | **0.135** | **171.010** | **31.778** | **1.16e^-73^** |
|  | CS | **-0.804** | **0.175** | **206.00** | **-4.608** | **7.12e^-06^** |
| Valence | (Intercept) | **2.797** | **0.148** | **116.319** | **18.881** | **3.21e^-37^** |
|  | CS | **0.449** | **0.148** | **206.000** | **3.039** | **.004** |

*Notes*. Ratings were given on a 7-point Likert scale, 1 being most negative/least arousing. *T*-statistics are evaluated for significance. A positive coefficient indicates higher ratings in the test condition than in the reference group. Effects with *p* ≤ .05 are marked in bold. Abbreviations: CS = conditioned stimulus, SE = standard error.

# P1 analysis

**Table S2. P1 Habituation: Linear Mixed Model Fixed Effects**

| Term | Estimate | *SE* | *df* | *t* | *p* |
| --- | --- | --- | --- | --- | --- |
| *Full sample* |  |  |  |  |  |
| (Intercept) | 2.580 | 0.342 | 132.814 | 7.554 | 6.09e^-12^ |
| CS | 0.216 | 0.298 | 2,339.320 | 0.726 | .468 |
| Hemisphere | **0.933** | **0.300** | **2,339.357** | **3.111** | **.002** |
| CS×Hemisphere | -0.172 | 0.419 | 2,338.892 | -0.409 | .682 |
| *SCR subsample* |  |  |  |  |  |
| (Intercept) | **3.618** | **0.466** | **55.347** | **7.766** | **2.02e^-10^** |
| CS | -0.166 | 0.357 | 1,264.588 | -0.466 | .635 |
| Hemisphere | -0.691 | 0.354 | 1,264.240 | -1.952 | .067 |
| CS×Hemisphere | -0.180 | 0.505 | 1,264.459 | -0.356 | .718 |

*Notes*. *T*-statistics are evaluated for significance. A positive coefficient indicates higher values in the test condition than in the reference group. Effects with *p* ≤ .05 are marked in bold. Abbreviations: CS = conditioned stimulus, SE = standard error.

**Table S3. P1 Acquisition: Linear Mixed Model Fixed Effects**

| Term | Estimate | *SE* | *df* | *t* | *p* |
| --- | --- | --- | --- | --- | --- |
| *Full sample* |  |  |  |  |  |
| (Intercept) | **3.448** | **0.282** | **82.073** | **12.237** | **3.39e^-20^** |
| CS | -0.122 | 0.143 | 10,601.558 | -0.851 | .395 |
| Hemisphere | **-0.314** | **0.143** | **10,603.152** | **-2.192** | **.028** |
| CS×Hemisphere | 0.024 | 0.203 | 10,602.095 | 0.119 | .905 |
| *SCR subsample* |  |  |  |  |  |
| (Intercept) | 3.568 | 0.406 | 42.337 | 8.784 | 4.33e^-11^ |
| CS | -0.239 | 0.199 | 5,621.295 | -1.202 | .241 |
| Hemisphere | **-0.387** | **0.199** | **5,622.109** | **-1.944** | **.048** |
| CS×Hemisphere | 0.301 | 0.282 | 5,621.566 | 1.068 | .279 |

*Notes*. *T*-statistics are evaluated for significance. A positive coefficient indicates higher values in the test condition than in the reference group. Effects with *p* ≤ .05 are marked in bold. Abbreviations: CS = conditioned stimulus, SE = standard error.

**Table S4. P1 Retrieval: Post-hoc CS Differences by SF × Hemisphere**

| SF | Hemisphere | Contrast | Estimate | *SE* | *df* | *z* | *p* | *d* |
| --- | --- | --- | --- | --- | --- | --- | --- | --- |
| *Full sample* | | | | | | | | |
| HSF | Left | (CS-) - (CS+) | 0.009 | 0.093 | 47080 | 0.100 | .920 | 0.004 |
| LSF | Left | (CS-) - (CS+) | **-0.268** | **0.094** | **47080** | **-2.862** | **.012** | **-0.326** |
| HSF | Right | (CS-) - (CS+) | -0.205 | 0.093 | 47080 | -2.189 | .058 | -0.244 |
| LSF | Right | (CS-) - (CS+) | -0.073 | 0.094 | 47080 | -0.784 | .433 | -0.116 |
| *SCR subsample* | | | | | | | | |
| HSF | Left | (CS-) - (CS+) | 0.144 | 0.127 | 24552 | 1.132 | .258 | 0.187 |
| LSF | Left | (CS-) - (CS+) | **-0.260** | **0.127** | **24552** | **-2.041** | **.041** | **-0.318** |
| HSF | Right | (CS-) - (CS+) | -0.207 | 0.128 | 24552 | -1.626 | .104 | -0.229 |
| LSF | Right | (CS-) - (CS+) | -0.045 | 0.127 | 24552 | -0.355 | .722 | -0.070 |

*Notes*. *T*-statistics are evaluated for significance. A positive coefficient indicates higher values in the test condition than in the reference group. Effects with *p* ≤ .05 are marked in bold. Abbreviations: CS = conditioned stimulus, HSF = high spatial frequency, LSF = low spatial frequency, SE = standard error, SF = spatial frequency.

# N1

**Table S5. N1 Habituation: Linear Mixed Model Fixed Effects**

| Term | Estimate | *SE* | *df* | *t* | *p* |
| --- | --- | --- | --- | --- | --- |
| *Full sample* |  |  |  |  |  |
| (Intercept) | **-1.758** | **0.370** | **131.207** | **-4.746** | **5.34e^-06^** |
| CS | 0.134 | 0.330 | 2,339.270 | 0.407 | .687 |
| Hemisphere | -0.176 | 0.329 | 2,338.063 | -0.536 | .569 |
| CS×Hemisphere | 0.024 | 0.470 | 2,338.770 | 0.050 | .957 |
| *SCR subsample* |  |  |  |  |  |
| (Intercept) | **-2.017** | **0.527** | **57.820** | **-3.828** | **3.20e^-04^** |
| CS | -0.008 | 0.419 | 1,264.584 | -0.019 | .984 |
| Hemisphere | 0.026 | 0.417 | 1,264.134 | 0.061 | .950 |
| CS×Hemisphere | -0.347 | 0.596 | 1,264.472 | -0.583 | .568 |

*Notes*. *T*-statistics are evaluated for significance. A positive coefficient indicates higher values in the test condition than in the reference group. Effects with *p* ≤ .05 are marked in bold. Abbreviations: CS = conditioned stimulus, SE = standard error.

**Table S6. N1 Retrieval: Linear Mixed Model Fixed Effects**

| Term | Estimate | *SE* | *df* | *t* | *p* |
| --- | --- | --- | --- | --- | --- |
| *Full sample* | | | | | |
| (Intercept) | **-2.113** | **0.261** | **79.558** | **-8.103** | **5.25e^-12^** |
| CS | -0.033 | 0.113 | 47,005.142 | -0.287 | .247 |
| SF | **0.625** | **0.113** | **47,005.389** | **5.511** | **3.58e^-08^** |
| Hemisphere | 0.169 | 0.113 | 47,004.925 | 1.493 | .206 |
| CS×SF | 0.140 | 0.160 | 47,005.239 | 0.873 | .359 |
| CS×Hemisphere | -0.008 | 0.160 | 47,005.123 | -0.047 | .194 |
| SF×Hemisphere | -0.112 | 0.160 | 47,004.925 | -0.701 | .055 |
| CS×SF×Hemisphere | -0.199 | 0.227 | 47,005.121 | -0.879 | .363 |
| *SCR subsample* | | | | | |
| (Intercept) | **-1.808** | **0.427** | **39.81** | **-4.232** | **1.32e^-04^** |
| CS | 0.009 | 0.162 | 24,509.05 | 0.054 | .949 |
| SF | **-0.713** | **0.161** | **24,509.17** | **-4.425** | **9.70e^-06^** |
| Hemisphere | 0.045 | 0.162 | 24,509.15 | 0.276 | .805 |
| CS×SF | -0.183 | 0.228 | 24,509.08 | -0.801 | .400 |
| CS×Hemisphere | 0.157 | 0.229 | 24,509.06 | 0.687 | .482 |
| SF×Hemisphere | 0.180 | 0.229 | 24,509.12 | 0.788 | .426 |
| CS×SF×Hemisphere | 0.068 | 0.323 | 24,509.04 | 0.212 | .809 |

*Note*. *T*-statistics are evaluated for significance. A positive coefficient indicates higher values in the test condition than in the reference group. Effects with *p* ≤ .05 are marked in bold. *Abbreviations*: CS = conditioned stimulus, SCR = skin conductance response, SE = standard error, SF = spatial frequency.

**Figure S1. SF differentiation in N1 amplitudes during the retrieval phase.** **a** Distribution of N1 amplitudes in response to each *SF* condition. Boxplots show median and interquartile range; raincloud plots display single-subject means per *SF* condition along with the smoothed distribution density; outlined diamonds indicate the overall condition mean. Stars and brackets mark significant post-hoc tests (****p* ≤ .001). **b** Average (top panel) and differential (LSF versus HSF, bottom panel) evoked time-series of responses in the channel cluster. The shaded area marks the N1 time-window. The topography depicts differential (LSF versus HSF) scalp signals. Channels that were used to extract the N1 are marked in black. *Abbreviations*: CS = conditioned stimulus, LSF = low spatial frequency, HSF = high spatial frequency, SF = spatial frequency.

# SCR

**Table S7. SCR Habituation: Linear Mixed Model Fixed Effects**

| Term | Estimate | *SE* | *df* | *t* | *p* |
| --- | --- | --- | --- | --- | --- |
| (Intercept) | **0.022** | **0.009** | **55.811** | **2.523** | **.015** |
| CS | 0.013 | 0.009 | 505.809 | 1.555 | 1 |

*Notes*. *T*-statistics are evaluated for significance. A positive coefficient indicates higher values in the test condition than in the reference group. Abbreviations: CS = conditioned stimulus, SCR = skin conductance response, SE = standard error.

**Table S8. SCR Acquisition: Linear Mixed Model Fixed Effects**

| Term | Estimate | *SE* | *df* | *t* | *p* |
| --- | --- | --- | --- | --- | --- |
| (Intercept) | 0.021 | 0.167 | 2,909.437 | 0.125 | .901 |
| CS | 3.03e^-04^ | 0.003 | 2,900.020 | 0.088 | .930 |

*Notes*. *T*-statistics are evaluated for significance. A positive coefficient indicates higher values in the test condition than in the reference group. Abbreviations: CS = conditioned stimulus, SCR = skin conductance response, SE = standard error.

**Table S9. SCR Retrieval: Post-hoc CS/SF Differences by Duration**

| Duration | Contrast | Estimate | *SE* | *df* | *z* | *p* | *d* |
| --- | --- | --- | --- | --- | --- | --- | --- |
| 100 ms | CS- – CS+ | 0.005 | 0.004 | 12166 | 1.233 | .218 | 0.259 |
| 1000 ms | CS- – CS+ | -0.007 | 0.004 | 12166 | -1.804 | .071 | -0.179 |
| 100 ms | HSF – LSF | 0.003 | 0.004 | 12126 | 0.951 | .342 | 0.182 |
| 1000 ms | HSF – LSF | -0.007 | 0.004 | 12126 | -1.798 | .072 | -0.255 |

*Notes*. *T*-statistics are evaluated for significance. A positive coefficient indicates higher values in the test condition than in the reference group. Abbreviations: CS = conditioned stimulus, SCR = skin conductance response, SE = standard error.

**Table S10. SCR – Retrieval: Linear Mixed Model Fixed Effects (without carryover trials)**

| Term | Estimate | *SE* | *df* | *t* | *p* |
| --- | --- | --- | --- | --- | --- |
| (Intercept) | **0.039** | **0.009** | **77.120** | **4.214** | **6.75e^-05^** |
| CS | -0.010 | 0.008 | 4,755.836 | -1.200 | .250 |
| SF | -0.012 | 0.008 | 4,755.305 | -1.400 | .190 |
| Duration | -0.014 | 0.008 | 4,756.100 | -1.786 | .066 |
| CS×SF | 0.017 | 0.012 | 4,755.348 | 1.481 | .151 |
| CS×Duration | 0.019 | 0.011 | 4,757.915 | 1.734 | .096 |
| SF×Duration | 0.013 | 0.011 | 4,756.654 | 1.173 | .250 |
| CS×SF×Duration | -0.022 | 0.016 | 4,757.252 | -1.374 | .169 |

*Note*. *T*-statistics are evaluated for significance. A positive coefficient indicates higher values in the test condition than in the reference group. Effects with *p* ≤ .05 are marked in bold. *Abbreviations*: CS = conditioned stimulus, SCR = skin conductance response, SE = standard error, SF = spatial frequency.

# P1 – SCR association

**Table S11. P1 – SCR association: Linear Mixed Model Fixed Effects**

| Term | Estimate | *SE* | *df* | *t* | *p* |
| --- | --- | --- | --- | --- | --- |
| (Intercept) | **-0.13** | **0.049** | **44.109** | **-2.643** | **.011** |
| Trial | **1.77e-04** | **4.18e-05** | **9,865.450** | **4.237** | **2.28e^-05^** |
| EEG | -0.022 | 0.053 | 5,866.942 | -0.426 | .781 |
| EEG×CS | **0.211** | **0.074** | **9,855.645** | **2.835** | **.047** |
| EEG×SF | -0.005 | 0.073 | 9,856.210 | -0.073 | .967 |
| EEG×Hemisphere | 0.019 | 0.033 | 9,829.114 | 0.589 | .719 |
| EEG×CS×SF | -0.175 | 0.105 | 9,857.765 | -1.677 | .240 |
| EEG×CS×Hemisphere | -0.096 | 0.046 | 9,849.477 | -2.055 | .159 |
| EEG×SF×Hemisphere | 0.005 | 0.046 | 9,854.808 | 0.114 | .937 |
| EEG×CS×SF×Hemisphere | 0.053 | 0.065 | 9,854.588 | 0.818 | .555 |

*Note*. *T*-statistics are evaluated for significance. A positive coefficient indicates higher values in the test condition than in the reference group. Effects with *p* ≤ .05 are marked in bold. *Abbreviations*: CS = conditioned stimulus, SCR = skin conductance response, SE = standard error, SF = spatial frequency.

**Table S12. Post-hoc tests P1 – SCR association: Linear Mixed Model Fixed Effects**

| CS | Std-β | Estimate | *SE* | *df* | *z* | *p* |
| --- | --- | --- | --- | --- | --- | --- |
| CS- | **0.028** | **0.0007** | **0.013** | **27.2** | **2.065** | **.039** |
| CS+ | 0.008 | 0.0004 | 0.013 | 25.7 | 0.579 | .562 |

*Notes*. *T*-statistics are evaluated for significance. A positive coefficient indicates higher values in the test condition than in the reference group. Effects with *p* ≤ .05 are marked in bold. Abbreviations: CS = conditioned stimulus, SCR = skin conductance response, SE = standard error.
